# Multimodality cardiac imaging of a double chambered right ventricle with intrapulmonary shunting: a case report

**DOI:** 10.1186/1756-0500-5-516

**Published:** 2012-09-22

**Authors:** Lan-Chau Kha, Alessandra Cassano-Bailey, Kelby Cleverley, Maneesh Sud, Jacek Strzelczyk, Davinder S Jassal

**Affiliations:** 1 Department of Radiology, University of Manitoba, Winnipeg, Manitoba, Canada; 2 Institute of Cardiovascular Sciences, St. Boniface General Hospital Research Centre, University of Manitoba, Winnipeg, Manitoba, Canada; 3 Section of Cardiology, Department of Internal Medicine, Rm Y3531, Bergen Cardiac Care Centre, St. Boniface General Hospital, University of Manitoba, 409 Tache Avenue, Winnipeg, Manitoba, R2H 2A6, Canada

**Keywords:** Echocardiography, Cardiac CT, Cardiac MRI, Cardiac catheterization, Double chambered right ventricle, Aortopulmonary shunt

## Abstract

**Background:**

Double chambered right ventricle (DCRV) is a relatively rare congenital heart disease, characterized by the abnormal division of the right ventricle into a high-pressure inlet and low-pressure outlet by anomalous muscle bundles. Extra-cardiac right-to-left shunts may present with clinical symptoms in adulthood and should be sought in patients with previous cavo-pulmonary shunt procedures.

**Case presentation:**

We report a case of DCRV in a 29 year old Caucasian male presenting in adulthood with a right-to-left shunt secondary to venous collaterals, following cavopulmonary anastomosis for congenital pulmonary atresia and hypoplastic right ventricle.

**Conclusion:**

Multimodality cardiac imaging using echocardiography, cardiac CT, cardiac MRI and cardiac catheterization is often required for complete characterization of complex congenital heart anomalies in adulthood.

## Background

Double chambered right ventricle (DCRV) is a congenital cardiac malformation in which the right ventricle is divided into a proximal high-pressure and distal low-pressure chamber by anomalous muscle bundles [1]. Echocardiography and cardiac magnetic resonance (CMR) imaging have increasingly become the modalities of choice for the non-invasive characterization of complex congenital heart lesions. We report a case of DCRV presenting in adulthood with a right to left shunt resulting from systemic venous to pulmonary venous collaterals that developed following superior vena cava to pulmonary artery shunt surgery for pulmonary atresia and hypoplastic right ventricle (RV).

## Case presentation

**Figure 1:**
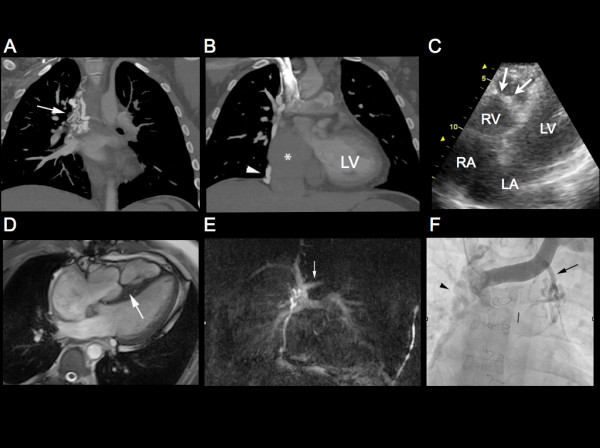
**(A) Maximum intensity projection pulmonary arterial phase CT images of the thorax reveal extensive venous collaterals arising from the superior vena cava (arrow), (B) which join to the inferior vena cava (arrowhead) and markedly dilated IVC and right atrium (star).** (**C**) An apical 4 chamber view on transthoracic echocardiography, demonstrating an echodense muscular band within the RV, consistent with a DCRV. (**D**) Balanced steady state free precession (B-SSFP) cine true axial reveal a thickened muscular band extending across the right ventricle (large arrow). (**E**) Subtracted MR pulmonary angiogram via right antecubital vein injection reveals a patent Glenn shunt and tortuous venous collaterals extending from the SVC to IVC as demonstrated on the CT examination. Note there is only very minimal reflux of contrast into the proximal left innominate vein (arrow). (**F**) Selective venogram of the left innominate vein demonstrates the large venous collateral draining into the left superior pulmonary vein (arrow) along with the previously demonstrated SVC-IVC venous collaterals (arrowhead).

A 29-year old Caucasian male presented with a one week history of increasing dyspnea on minimal exertion and two syncopal episodes. His past history was notable for pulmonary atresia, a hypoplastic RV and a secundum atrial septal defect (ASD) at birth, for which he underwent closed pulmonic valvotomy and creation of a central aortopulmonary (AP) shunt on day 2 of life. He subsequently underwent a classic Glenn shunt, ligation of the central AP shunt and finally closure of the ASD secundum. He was lost to follow-up until his current presentation. On physical examination, the patient was afebrile with a normal pulse and blood pressure. Peripheral O_2_ saturation was 84% on room air. The jugular venous pulsation was elevated at 6 cm above the sternal angle. Auscultation revealed a grade 3/6 systolic murmur over the left lower sternal border and bibasilar crackles. A room air arterial blood gas demonstrated a pH of 7.51, PaO_2_ of 64 mmHg, PaCO_2_ of 28 mmHg, and HCO_3_^-^ of 25 mmol/L. Electrocardiogram revealed evidence of right atrial enlargement with nonspecific ST-T wave changes across the precordial leads. Computed tomography (CT) of the chest revealed no evidence of a pulmonary embolism. Of note, the right atrium and inferior vena cava (IVC) were markedly dilated and several venous collateral vessels were observed between the superior vena cava (SVC) and IVC (Figures 1A-B). Transthoracic and transesophageal echocardiography (TTE and TEE) revealed evidence of a double chambered right ventricle with a peak systolic gradient of 50 mmHg across a prominent moderator band (Figure 1C). There was no evidence of obstruction noted across the pulmonary outflow tract. The left ventricular systolic function was within normal limits with no valvular abnormalities. Following administration of agitated saline contrast from the left antecubital vein, there was evidence of right-to-left shunting. The site of shunting could not be identified on TTE nor TEE. Further imaging with CMR revealed a hypertrophied muscle bundle dividing the RV into two chambers with a systolic dephasing jet across this band corresponding to the region of flow acceleration detected on TTE (Figure 1D), confirming the diagnosis of a DCRV. The CMR pulmonary angiogram (Figure 1E) confirmed venous collaterals extending from the SVC to IVC, with no explanation for the right-to-left shunting. Right heart catheterization revealed a large venous collateral arising from the left innominate vein draining into a segmental branch of the left superior pulmonary vein (Figure 1F).

## Discussion

Double chambered right ventricle (DCRV) is an uncommon congenital cardiac anomaly comprising approximately 1% of all cardiac malformations and approximately 2% of all congenital cardiac lesions detected in adults [1]. In DCRV, the apical trabecular portion of the RV is divided by anomalous muscular bands into a proximal high-pressure inlet and distal low-pressure outlet chamber [1]. Other cardiac anomalies associated with DCRV include ventricular septal defects, Tetralogy of Fallot and pulmonary atresia [1].

Pulmonary atresia with an intact ventricular septum (PAIVS) is a spectrum of duct-dependent cyanotic congenital lesions, which include atresia of the pulmonary valve, varying degrees of right ventricular and tricuspid valve (TV) hypoplasia, along with anomalies of the coronary circulation [2]. Surgical treatment for PAIVS commonly occurs in two stages. The first stage consists of transpulmonary valvotomy with possible creation of a systemic-pulmonary artery shunt to relieve the duct-dependent circulation, promote forward blood flow and allow for growth of the hypoplastic RV. The second and often definitive repair stage, consists of an anatomic biventricular repair, a one-and-a-half ventricular repair or a Fontan-type procedure depending on the degree of residual hypoplasia of the RV and TV [2]. Our patient underwent repair of his PAIVS and hypoplastic RV with a closed pulmonic valvotomy and creation of central AP shunt, followed by a definitive one-and-a-half ventricular repair consisting of a bidirectional superior vena cava-pulmonary artery (Glenn) shunt, ASD closure and central AP shunt closure.

Up to 33% of cavo-pulmonary artery shunts develop pulmonary AV fistulas and venous collaterals between the high pressure SVC and low pressure IVC system [3]. These venous collaterals and arteriovenous malformations result in significant right-to-left shunting causing systemic arterial desaturation, which can necessitate coil embolization or surgical ligation to improve arterial oxygen saturation [4].

Although multiple collaterals from the SVC and IVC were demonstrated on CT and CMR pulmonary angiograms in our patient, interestingly, a systemic venous-to-pulmonary venous collateral, causing right-to-left shunt was also identified on transthoracic echocardiography and likely a major factor in his hypoxia. Our patient developed these collaterals likely due to the obstruction noted across the moderator band in the DCRV. The venous collateral shunt arising from the left innominate vein draining into a segmental branch of the left superior pulmonary vein was ultimately identified with right heart catheterization and not CT or CMR pulmonary angiograms. This may have been due to the administration of contrast though the right antecubital vein in both studies resulting in lack of opacification of the left innominate vein.

Both echocardiography and CMR are valuable imaging modalities capable of characterizing right ventricular morphology and quantitative function in DCRV, without the risk of radiation exposure [4,5]. In the setting of a prior cavopulmonary shunt, however, the detection of venous collateral channels and arteriovenous malformations may be limited due to technical approach and inadequate measures for quantifying extra-cardiac shunts. Right heart angiography remains essential in identification and quantification of such lesions, as demonstrated in the current case.

One of the problems and challenges in the management of adults with congenital heart disease is the large number of different anomalies that the clinician may encounter. In patients with DCRV and PAIVS, venous collaterals and arteriovenous malformations can result in significant right-to-left shunting causing systemic cyanosis in adults. Transthoracic echocardiography with agitated saline contrast should be administered through both the right and left antecubital veins for the identification of a right-to-left shunt in this patient population. Following the TTE, clinicians should have a low threshold in using complementary imaging techniques to further delineate the exact location of the shunt, prior to considering either percutaneous or surgical correction of the disease.

## Conclusion

Multimodality cardiac imaging using echocardiography, cardiac CT, cardiac MRI and cardiac catheterization is often required for complete characterization of complex congenital heart anomalies in adulthood.

## Consent

A written informed consent was obtained from the patient for publication of this case report and all accompanying images.

## Competing interests

No competing issues to report.

## Authors’ contributions

LK, AC, and MS contributed to the acquisition of data and interpretation of the case report. LK, AC, KC, MS, JS and DJ contributed to the drafting of the manuscript, including the figures. All authors read and approved the final manuscript.
